# Retraction: Up-regulation of microRNA let-7c by quercetin inhibits pancreatic cancer progression by activation of Numbl

**DOI:** 10.18632/oncotarget.28751

**Published:** 2025-06-25

**Authors:** Clifford C. Nwaeburu, Natalie Bauer, Zhefu Zhao, Alia Abukiwan, Jury Gladkich, Axel Benner, Ingrid Herr

**Affiliations:** ^1^Team Molecular OncoSurgery, Section Surgical Research, Department of General, Visceral and Transplantation Surgery, University of Heidelberg, Heidelberg, Germany; ^2^Department of Biostatistics, German Cancer Research Center (DKFZ), Heidelberg, Germany

**This article has been retracted:** Oncotarget has completed its investigation of this article, which determined that many instances of image overlap and duplication compromise the integrity of the presented data and findings. Wound healing assay Images from different treatment groups of different cell lines were the result of manipulations with a few initial images.

Specifically, in Figure 3D, the 0-hour timepoint images one and two appear to be duplicates of images two and four, respectively. Furthermore, three images of AsPC-1 cells in Figure 3D at the 24-hour and 48-hour timepoints seem to be duplicates of three images of AsanPaCa cells found in Supplementary Figure 1E. Upon further review of Supplementary Figure 1E, we identified internal duplications at the 0-hour timepoint for both AsanPaCa cells (images 1 and 3 are duplicates of images 2 and 4) and PANC 1 cells (the top three images are duplicates). In addition, we have found duplications within the colony formation assay images presented in Figure 3A–3C. In Figure 3A, the last two images, representing different treatments, are duplicates. Moreover, the third image of Asan PaCa cells in Figure 3B appears to be a duplicate of the fourth image of PANC 1 cells in Figure 3C.

First author Clifford Nwaeburu provided corrected files for a new Supplementary Figure 1 and Figure 3, but these did not resolve all the issues noted. Corresponding author Ingrid Herr contacted Onoctarget regarding the remaining image duplications and requested retraction of the article. Based on these circumstances, the Editorial decision was made to retract this paper. All authors except the first author, Clifford C. Nwaeburu, agreed with the decision.

Original article: Oncotarget. 2016; 7:58367–58380. 58367-58380. https://doi.org/10.18632/oncotarget.11122

